# Utility of a Single-Tube, Six-Color Flow Cytometry Panel for the Diagnosis of Myelodysplastic Syndrome: Experience of a Tertiary Care Centre in India

**Published:** 2018-01-01

**Authors:** R. Gupta, K. Rahman, M.K. Singh, S. Kumari, G. Yadav, S. Nityanand

**Affiliations:** Department of Hematology, Sanjay Gandhi Post Graduate Institute of Medical Sciences, Lucknow, India

**Keywords:** MDS, Flow cytometry, FCM score, India

## Abstract

**Background: **Diagnosis of myelodysplastic syndromes (MDS) is challenging in the presence of morphological mimickers. Flow cytometric immunophenotyping (FCI) has been added to the diagnostic armamentarium, but its use in clinical practice is variable.

**Materials and Methods: **Bone marrow aspirate samples from 54 patients with a clinical and/or morphological suspicion of MDS were subjected to FCI using a single-tube, 6-colour panel comprising of monoclonal antibodies against CD13, CD11b, CD16, CD34, CD45 and CD56. Analysis was centered on the abnormal maturation pattern of granulocytes, blast percentage (≥3%) and ratio of side scatter peak channel value (SSC-PCV) of granulocytes and lymphocytes. Each of these parameters was assigned a score of 1. Overall sensitivity and specificity of this panel was ascertained to differentiate cytopenia/s of MDS from non-MDS cases.

**Results: **Forty MDS and 14 non-MDS cases were diagnosed based on morphology and cytogenetic results. Twenty control samples were also processed simultaneously for FCI to assign the cutoff for various flow cytometric parameters. A score ≥2 was defined as positive for MDS. Hypogranularity was present in 62.5% cases of MDS. The median SSC-PCV of granulocytes and lymphocytes was 6.16 in the MDS group, 7.9 in the non-MDS group and 8.90 in the control group (p <0.05). This cut-off value of 6.16 had a specificity of 92.5% based on the ROC curve analysis. Abnormal granulocyte maturation patterns for CD13/16, CD13/11b and CD11b/16 dot plots were observed in 95.3, 69.8 and 74.4% cases, respectively. The overall sensitivity and specificity of the panel was found to be 87.5% and 64.2%, respectively.

**Conclusion: **FCI is now an important tool for diagnostic workup in patients presenting with persistent cytopenia with or without morphological evidence of dyspoiesis. Inclusion of objective parameters like SSC-PCV would also reduce inter-lab variability in MDS diagnosis.

## Introduction

 Myelodysplastic syndrome (MDS) is a heterogeneous group of clonal hematopoietic stem cell disorder characterized by morphological dysplasia and ineffective erythropoiesis^   1 ^. Diagnosis is established primarily on the basis of morphology and cytogenetics. However, the dysplastic features are not unique to MDS and are subject to inter-observer variability, especially in low grade MDS. Cytogenetic abnormalities are detected in only 50% individuals and might not assist in confirming the diagnosis^2^^,^^3^. Flow cytometric immunophenotyping (FCI) provides an objective evidence of dysplasia and has proven to be a powerful diagnostic tool in documenting dysplasia even in the absence of appreciable dysplastic morphological features or non-informative cytogenetics^4^^,^^5^. The International working group has recommended the use of FCM to establish maturation abnormalities since it can identify specific aberrations in the precursor cells, maturing myelo-monocytic cells as well as in erythroid lineage^6^^,^^7^. The European Leukemia Net workshop held in 2008, sought to standardize methods, antibody combinations and interpretations of FCM of bone marrow in MDS^6^ and concluded that despite strong evidence for an impact of FCM in myelodysplastic syndromes, prospective validation of markers is still required. Recently Dongen V JJ et al. have proposed the euroflow panels for AML/MDS, which incorporates an exhaustive list of antibodies in four tubes with 8- colour antibody combinations. This panel elaborately evaluates the blasts, maturation profiles of all myeloid lineages and aberrantly expressed lymphoid markers in MDS^8^. 

Despite all these developments, there are no consensus guidelines on the minimal panel of antibodies required to ascertain the diagnosis of MDS with a good sensitivity and specificity. Thus in this study, we evaluated the utility of a single-tube, 6-color panel for the diagnosis of MDS and differentiating it from other pathological states presenting with persistent cytopenias. 

## Materials & Methods

**Patients & bone marrow examination**

 This was a prospective study done over a period of 33 months in the department of Haematology, SGPGI, Lucknow, India. Bone marrow aspirate samples from the patients with a working diagnosis of MDS (based on clinical and morphological finding) were subjected to FCI. All clinical and laboratory details of patients were collected. A written informed consent was taken from each patient. The study was approved by the institute’s Ethics Committee. 

Morphological examination was done on May-Grünwald Giemsa (MGG) stained peripheral blood and bone marrow aspirates. The smears were reviewed by two hematopathologist (RG & KR) and a diagnosis of MDS was made based on WHO 2008 classification. The terminologies of different categories were further amended based on WHO 2016 classification. Perl’s stain for iron stores and ringed sideroblasts were performed on all cases. 

**Flow cytometric immunophenotypic analysis**

 FCI was carried out on ethylene diamine tetra acetic acid (EDTA) anticoagulated bone marrow aspirate samples. A stain-lyse-wash protocol was used. Antibodies used were in a single-tube, six -color combination with following antibody-fluorochrome combination: CD16-FITC, CD11b-PE, CD34-PerCP CY5.5, CD13-PeCy7, CD56-APC, CD45-APCH7* (Beckton Dickinson Biosciences, San Jose, CA, USA)*. Briefly 100-200 micro litre of sample were incubated with pre-titrated amount of antibody cocktail for 20 minutes followed by red cells lysis with commercial lysing agent. This was followed by washing and a final suspension in 0.5ml of paraformaldehyde. Acquisition and analysis were done on BD FACS canto II platform using BD FACS Diva v8.0 software. A minimum of 100, 000 CD45 positive events were analyzed in each case. Analysis was predominantly focused on the maturation patterns in the myeloid series cells along with proportion and abnormalities in the CD34 positive compartment. The mean fluorescent intensities (MFI) of CD45 and side scatter peak channel values (SSC PCV) in the lymphoid cells and maturing myeloid cells were also evaluated.

In addition to the suspected MDS cases, 20 bone marrow aspirate samples from immune thrombocytopenia, aplastic anemia and lymphoma staging patients were taken as control to assign the cutoff for all above-mentioned parameters.

**Flow cytometry scoring**

A score of 1 was given to each of the 5 features evaluated, namely i) hypogranulatrity based on reduced scatter on the side scatter (SSC) vs CD45 plot, the side scatter peak channel values (SSC PCV) ratio of the granulocytes and lymphoid cells (Gra/Ly ratio), ii) aberrant maturation pattern of myeloid series cells as assessed on iii) CD13 vs CD16 iv) CD13 vs CD11b & v) CD11b vs CD16 scatter plots ; and the proportion of blast based on CD34/SSC dot plot amongst all nucleated cells. Median values were calculated for each of the numerical parameter, and a cutoff was assigned based on the results of the control group. Each of the abnormal parameters was assigned a score of 1, thereby achieving a maximum score of 5. A cutoff of score of ≥ 2 was considered as positive for MDS, whereas 0/1 was considered negative since none of our controls had a score greater than 1. Abnormalities in the maturation pattern were analyzed based on the patterns documented in the control group and literature^4^.

**Statistical analysis**

The mean, median and ratio for different parameters were calculated from the data. The difference among groups and correlation studies were analyzed by analysis of variance and Pearson correlation. ROC curve analysis was performed to ascertain a cutoff value for the Gra/Ly SSC –PCV ratio. Results with a p-value of 0.05 or less (p≤ 0.05) was considered statistically significant. 

## Results

**Patients’ characteristics**

A total of 54 patients and 20 controls were evaluated in the study. The median age of the patients was 59 years (range 3-80 years). Pancytopenia was observed in 52.6% (n = 30) patients at presentation, whereas bicytopenia and isolated anemia were seen in 37% (n = 20) and 7% (n = 4) cases, respectively. The baseline characteristics of the patients are shown in Table 1. 

Forty (40/54, 74%) patients were diagnosed to have MDS based on the morphological and cytogenetic findings. They were further morphologically classified as per the new WHO 2016 into MDS-SLD (n=5), MDS – MLD (n=14), MDS-MLD with RS (n=3), MDS-EB-1 (n=10), MDS-EB (n=8). One of the patients (1/54) had unexplained cytopenias, minimal morphological dysplasia & normal cytogenetics, and was thus considered to have idiopathic cytopenia of undetermined significance. The remaining thirteen (13/54) patients, whose samples were subjected to FCI had a final diagnosis of nutritional anemia (n= 6), hypoplastic anemia (n= 3), low grade lymphoma infiltration (n=1), PRCA (n=1) and granulomatous inflammation (n= 2). 

**Table 1:** Patient characteristics of patients diagnosed as MDS (n = 40)

**Total Patients (n)**	** 40**
Gender Male Female	27 13
Age (years) Median (Range 3-80 )	59
Age group <40 years 40-60 years >60 years	7 23 10
Cytopenias at presentation Isolated thrombocytopenia Anemia Bicytopenia Pancytopenia	0 04 20 30
Hemoglobin (g/dL) Median (Range)	6.9 (2.4-12)
Total Leukocyte counts/TLC (x10^9^/ L) Median (Range)	3.9 (0.9- 57.9)
Platelet counts (x10^9^/ L) Median (Range)	50.0 (8-438)
WHO diagnosis MDS - SLD MDS - MLD MDS - EB I MDS - EB II	5 17 10 08

**Flow cytometry analysis**

The results of flow cytometry were compared between the MDS group (n = 40 cases), non-MDS group (n=13) and control group (n = 20) to assess the utility of the proposed parameters in confirming the diagnosis (Table 2). 

**Figure 1:**
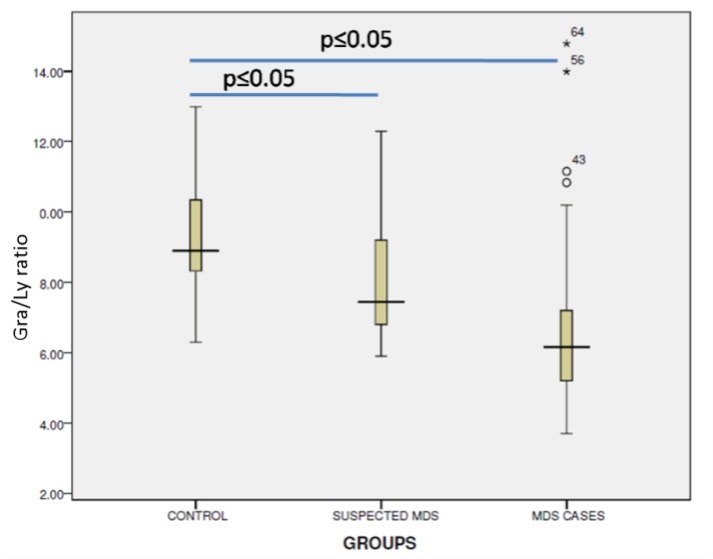
Box plot showing the distribution and difference in the Gra/Ly ratios of the SSC peak channel value of the granulocytes and lymphocytes in the three groups viz, control group, Non MDS group and the MDS group.

Overall, the concordant diagnosis of MDS by morphology and flow cytometry was obtained in 35/40 (87.5%) patients. Amongst this category of MDS, hypogranularity, as deduced by the reduced side scatter in CD45 vs SSC plot, was present in 62.5% cases. The median Granulocyte/Lymphocyte ratio derived from the SSC-PCV of granulocytes and lymphocytes was 6.16 in the MDS group, 7.9 in the non-MDS group and 8.90 in the control group. This cut-off value of 6.16 was found to be statistically significant among the three groups (p <0.05; Table 2; Figure 1). 

Gra/Ly ratio less than 6.16, as a single altered parameter, was found to have a very high specificity of 92.6% in favoring a diagnosis of MDS. Peripheral blood blast percentage equal or greater than 3% was set as cutoff for score 1, based on analysis of control samples.

**Table2:** Baseline characteristics and results of flow cytometry analysis amongst the three groups

	**MDS CASES (n = 40)**	**NON MDS CASES ( n =14)**	**CONTROL (n = 20)**
Median Age in Years (Range)	59.0 (3-80)	52.0 (7-62 )	49.0 (4-75)
Morphological categories	SLD (5) MDL (17) EB 1 (10) EB 2 (8)	Hypoplastic anemia (3) Granulomas (2) Nutritional (6) Lymphoma staging (1) PRCA (1) Unexplained cytopenia (1)	Aplastic/Hypoplastic (4) ITP (4) NHL staging (4) PUO with cytopenias (2) PRCA (1) CMPD (2) Metastasis (1) Post Chemotherapy (2)
Presence of hypogranularity	27 (62.8%)	5 (38.4%)	4 (19%)
SSC PCV Granulocytes (Gr)	107098	108020	128890
SSCPCV Lymphocyte (Ly)	16084	14197	13490
Gra/ Ly * (range)	6.16 (3.7-14.8)	7.45 (5.3-12.3)	8.90 (6.3-11.09)
Abnormal maturation pattern CD13/16 pattern CD13/11b pattern CD11b/16 pattern	41 (95.3%) 30 (69.8%) 32 (74.4%)	1 (7.6%) 0 1 (7.6%)	1 (4.8%) 0 0
Blast % Mean Median	3.0 1.6	1.12 0.9	0.7 0.7

* p Value is significant among three groups for these parameters, p<0.05, SSC-side scatter, PCV- Peak channel value. PRCA –pure red cell aplasia, ITP – Immune thrombocytopenic purpura, NHL- Non hodgkin lymphoma, PUO-pyrexia of unknown origin, CMPD – chronic myeloproliferative disorder

**Figure2:**
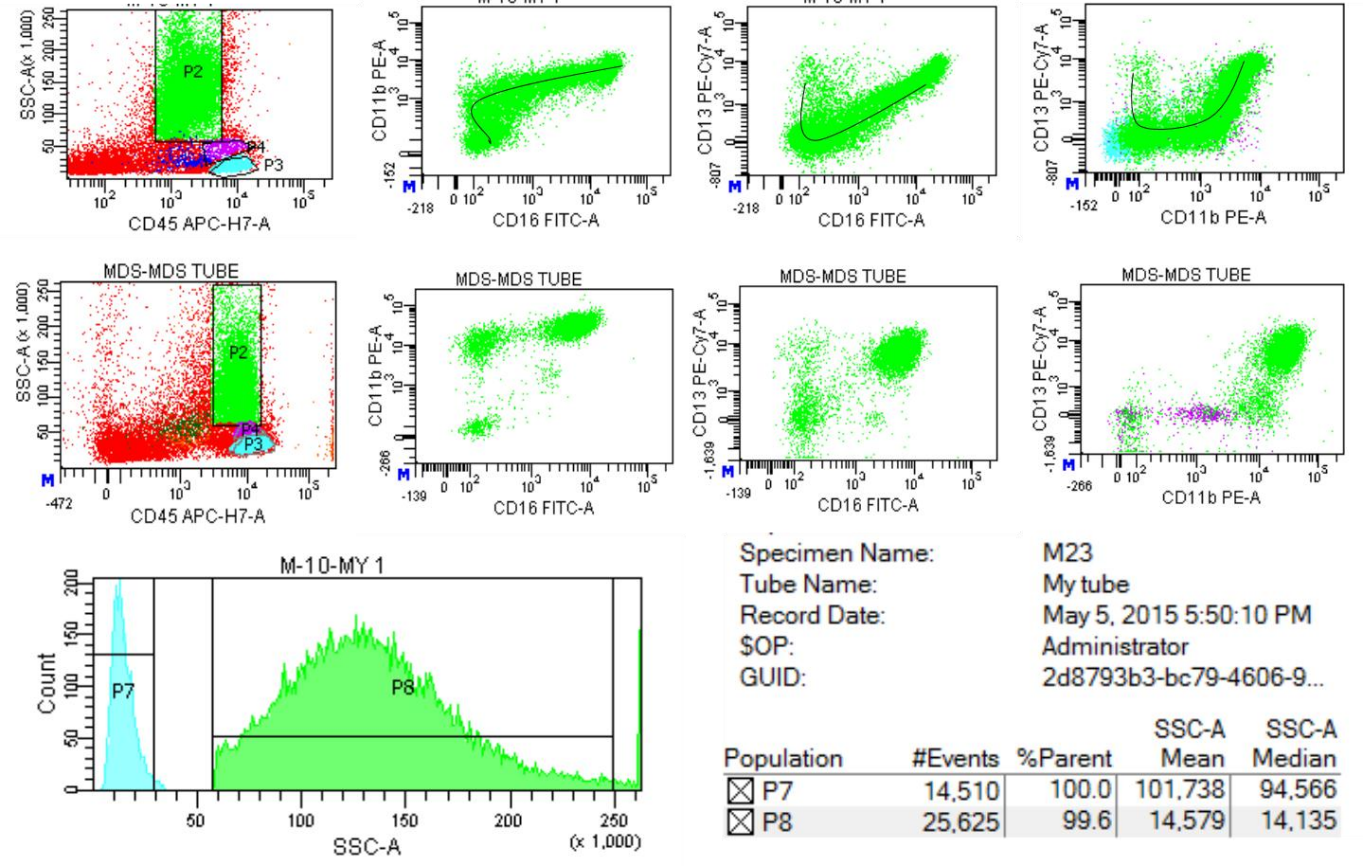
Flow cytometry dot plots showing normal myeloid maturation pattern using CD13, CD16 and CD11b (top row). The middle row is from a representative sample of proven MDS, where all three patterns are distorted. The last row of the plot shows the SSC peak channel value of granulocyte (P8) to lymphoid (P9) and derivation of the Gra/Ly ratio using FACS diva software v8.

Presence of abnormal maturation pattern of granulocytes as reflected by the CD13/16, CD13/11b and CD11b/16 dot plots was observed in 95.3%, 69.8% and 74.4% cases, respectively (Figure 2). 

A minimum deviation of half log intensity from the defined normal form was considered positive for these antigens. CD56 expression in the myeloid blasts was seen in only two cases of RAEB 1. A false negative FCI was observed in 5/40 cases, which included cases of RA (n=1), RCMD (n=3), and a single case of RAEB-1. False positive FCI was seen in 5 cases, which included patients with lymphoma infiltration (n= 1), granulomatous pathology and 2 cases of nutritional anemia. One of the cases, with minimal dyspoiesis morphologically, had a FCI score of 3, thereby favoring a diagnosis of MDS. The overall sensitivity and specificity of the panel was found to be 87.5% and 64.2%, respectively. The positive predictive value and negative predictive value were 88% and 64.2%, respectively.

## Discussion

 Myelodysplastic syndrome is a heterogeneous group of clonal haematological disorder in terms of morphology, cytogenetics and clinical outcome. In developing countries like India, the diagnosis is largely based on the presence of morphological dysplasia and cytogenetics, if available. However, the presence of morphological mimickers and presence of clonal abnormalities in a limited number of patients may pose a diagnostic challenge. Moreover, cytogenetic studies are labor intensive and time consuming, as well as requiring a certain level of scientific expertise. On the other hand, flow cytometric immunophenotyping, is a highly sensitive and reproducible method for quantitatively and qualitatively evaluating hematopoietic cell abnormalities. Studies have shown a high correlation between FCI abnormalities in MDS patients^4^^,^^5^^,^^9^^,^^10^^,^^11^^,^^12^. The major limitation in applying flow cytometry in routine diagnosis is the extensive panel required to document the dysplastic changes in different hematopoietic cell lineages, lack of uniformity in antibody panels and analysis strategies across various centers performing FCI for MDS^7^^,^^10^^, ^^12^. The aim of this study was to ascertain the value of a single-tube, 6-colour panel in distinguishing MDS from other causes of cytopenia(s). By the use of this panel, a maximum score of 5 was obtained. Otherwise, it is recommended to favor a diagnosis of MDS when 3 or more abnormalities are detected by FCI^4^^, ^^5^. Since majority of our non-MDS cases as well as controls had a score of 0/1, thus a cutoff of 2 was considered optimal for MDS diagnosis. In true MDS cases, the incidence of hypogranularity was 62.5%, which is similar to the observations made by other authors^4^^,^^11^^,^^13^. The SSC PCV of the granulocytes and lymphocytes were computed using the software^   15 ^, and their ratio was calculated. We observed that a Gra/Ly ratio cutoff of 6.16 was a strong predictor of hypogranularity with a sensitivity of 90.4% and specificity of 86.6%. Similar parameter has been implemented by Kern et al. in their study cohort to delineate MDS-related pancytopenia from other etiologies. They observed a cutoff of 6 to be statistically important^   12 ^. The usefulness of this particular parameter has also been validated by Porta et al. ^14^, wherein the Gra/LySSC- PCV ratio was found to be reduced in their MDS patients (median of 6.46, range 2.05–18.5) compared to the controls (7.98, range 4.40–15.96), which was statistically significant (p<0.001). However, this parameter has to be applied in conjunction with other findings since a ratio less than 6.16 was also observed in 2 non-MDS (false positive) cases. 

Other parameters evaluated included the myeloid maturation pattern and myeloblasts %. CD13/CD16, CD11b/CD13 and CD11b/CD16 dot plots revealed distorted patterns in 95%, 70% and 74% cases, respectively, which are in concordance with documented literature^4^^,^^11^^,^^13^. CD34-positive progenitor component is definitely disturbed in MDS. Morphological evaluation of blasts in cases of MDS can be tricky at times since there is extensive inter-observer variability and the myeloblasts mimicking erythroblasts and monocytoid precursors in certain cases of MDS. Though the blast percentage is comparatively lower than their morphological counterparts as detected by FCI, the presence of aberrancies assist in confirming the diagnosis. Aberrant expression of CD56 was observed in the myeloblasts in 2/40 cases of MDS. 

As already mentioned, besides antibodies used in our panel, other authors have also proposed certain antibody combination which is useful in predicting MDS. Ogata et al. have proposed a combination of four parameters: the percentage of CD34+ myeloid progenitor cells in the bone marrow, the frequency of B-cell precursors within the CD34+ compartment, CD45 expression on myeloid progenitors relative to CD45 expression on lymphocytes, and the evaluation of neutrophil granularity by comparison to SSC on lymphocytes. The diagnostic sensitivities were 65% and 89% and the specificities were 98% and 90% for a score ≥2 in the Japanese and Italian cohorts, respectively. The further addition of three other parameters, namely, the expression of CD11b, CD15, and CD56 on CD34+ myeloblasts, did not improve the diagnostic power. Our panel partly evaluated the proposed parameters, of which percentage of myeloid blasts and Gra/Ly ratio was found useful in establishing the diagnosis of MDS. On the contrary, Chung JW et al. have proposed the use of a combination of CD15/CD10, CD64/CD33, CD16/CD13 or CD11b flow cytometric granulocyte panels. Maximum sensitivity rates for 3 panels were 89.7% as CD15/CD10/CD45 panel, CD64/CD33/CD45 panel and CD16/CD13 or CD11b/CD45 combinations were employed^15^.

However, we documented certain limitations of the present proposed panel. These include the lack of additional blast/ lineage/ hematogones defining markers like CD10, CD19 and CD117. It had already been observed that the lymphoblasts progenitors, identified by very low SSC & dim to negative CD45 expression in the CD45 versus SSC plot appeared to be reduced in patients with MDS, and in absence of CD19/CD10 in our panel, this parameter could not be evaluated. Moreover, CD56 aberrant expression in myeloblasts was seen in only 5% cases of MDS, thereby reducing its utility was considered as a potential marker for MDS, particularly in evaluating dysplasia in granulocytes and blasts. Incorporation of lineage infidelity markers like CD2 and CD7 would have increased the specificity of the panel.

## CONCLUSION

 In conclusion, though a combination of morphology and cytogenetics are the key determinants of MDS diagnosis, flow cytometry appears to be a powerful tool in clinical practice for patients with unexplained cytopenias. A definite diagnosis of MDS can be made with the help of a limited FCI panel, even in the presence of non-contributory cytogenetics and subtle morphological dysplasia. The panel used in our study may not be an ideal tool, but it certainly helps in delineating the MDS cases from non-MDS cases without adding very high costs to the services. The incorporation of additional markers would definitely assist in the more precise diagnosis of patients. In the absence of a unified flow cytometric panel for MDS diagnosis, individual laboratories are recommend to define their own cut-off values based on their panel and instrument set up. 
